# Higher Levels of Low Molecular Weight Sulfur Compounds and Homocysteine Thiolactone in the Urine of Autistic Children

**DOI:** 10.3390/molecules25040973

**Published:** 2020-02-21

**Authors:** Paulina Gątarek, Angelina Rosiak, Kamila Borowczyk, Rafał Głowacki, Joanna Kałużna-Czaplińska

**Affiliations:** 1 Institute of General and Ecological Chemistry, Faculty of Chemistry, Lodz University of Technology, Zeromskiego 116, 90-924 Lodz, Poland; gatarekpaulina@gmail.com (P.G.); angelina.rosiak@p.lodz.pl (A.R.); 2 Department of Environmental Chemistry, Faculty of Chemistry, University of Lodz, 91-403 Lodz, Poland; kamila.borowczyk@chemia.uni.lodz.pl (K.B.); rafal.glowacki@chemia.uni.lodz.pl (R.G.)

**Keywords:** autism, homocysteine thiolactone, cysteine, cysteinylglycine

## Abstract

In this study, the levels of concentration of homocysteine thiolactone (HTL), cysteine (Cys), and cysteinylglycine (CysGly) in the urine of autistic and non-autistic children were investigated and compared. HTL has never been analyzed in autistic children. The levels of low molecular weight sulfur compounds in the urine of both groups were determined by validated methods based on high-performance liquid chromatography with spectrofluorometric and diode-array detectors. The statistical data show a significant difference between the examined groups. Children with autism were characterized by a significantly higher level of HTL (p = 5.86 × 10^−8^), Cys (p = 1.49 × 10^−10^) and CysGly (p = 1.06 × 10^−8^) in urine compared with the control group. A difference in the p-value of <0.05 is statistically significant. Higher levels of HTL, Cys, and CysGly in the urine of 41 children with autism, aged 3 to 17, were observed. The obtained results may indicate disturbances in the metabolism of methionine, Cys, and glutathione in some autistic patients. These preliminary results suggest that further research with more rigorous designs and a large number of subjects is needed.

## 1. Introduction

Autism spectrum disorder (ASD) is a neurodevelopmental disorder characterized by complex etiology, impaired social communication and interaction, and also restricted repetitive behavior [1]. Many scientists focus on the determination of metabolites in the urine and blood of ASD children. The identification of an abnormal metabolite in the blood and urine of individuals with ASD has the potential to aid diagnosis and identify ASD subtypes, which would result in the greater individualization of therapeutic decisions. Compared with blood, the collection of urine samples is relatively simple, convenient, and non-invasive [2,3,4].

Diseases caused by disturbances in amino acid metabolism can cause brain damage and a delay in mental development. Amino acids passing through the blood–cerebrospinal fluid barrier, such as phenylalanine, tyrosine, and tryptophan, are precursors of neurotransmitters regulating the functioning of the central nervous system that affect the emotional state and mood among others. In addition, the level of amino acids excreted with the urine provides important information about not only the dietary factors but also the factors connected with the digestive system affecting the occurrence and severity of autism symptoms [5,6]. Compounds containing a thiol group in their structure, such as homocysteine (Hcy), cysteine (Cys), glutathione (GSH), cysteinylglycine (CysGly), and γ-glutamylcysteine (γ-GluCys), play a significant role in living organisms. They are involved in numerous biochemical processes at the cellular level and determine the proper functioning of organisms. As a result, the disorder of metabolism often leads to diseases in the human body [7,8,9]. Homocysteine thiolactone (HTL) is a well-known modifying factor of proteins but its role in the pathogenesis of different diseases is poorly recognized. Only associations of HTL with cardiovascular diseases are described in the literature [10,11]. Moreover, very preliminary studies have shown that urinary HTL can act as a risk predictor of acute myocardial infarction in patients with coronary artery disease [12].

In the case of autistic children, different metabolic disorders are discovered. These include phenyloketonuria, histidinemia, urea cycle disorder, adenylosuccinate lyase deficiency, 5′-nucleotidase superactivity, creatine deficiency syndromes, and also disorders related to the metabolism of cholesterol, amino acid, cofactor (vitamin), γ-aminobutyric acid, pyrimidine, and purine metabolism [13,14]. Several studies suggest that Hcy, which is produced during methionine metabolism in the body and expelled in urine and plasma, provides essential information on the diet and the alimentary system. There are studies on the neurotoxicity of Hcy that show that Hcy can induce neuronal damage and cell loss through excitotoxicity as well as apoptosis [15,16].

Chromatographic techniques play an essential role in the determination of the concentration of low molecular weight sulfur compounds in biological samples,. There are many methods in the literature dedicated to hydrophilic thiol amino acids using high-performance reverse phase liquid chromatography (RP-HPLC) with spectrophotometric detection (UV–Vis) and spectrofluorometric detection [17,18]. HTL was determined with the use of different methods based on separation techniques. One of them is based on the solid phase extraction, the column derivatization of HTL with *O*-phthaldialdehyde, and spectrofluorometric detection [19]. The second is based on capillary electrophoresis capillary electrophoresis (CE) and ultra-violet detection (UV) [20]. Additionally, the gas chromatography/mass spectrometry method was applied in the analysis of human urine for HTL [21].

In this paper, the relationship between low molecular weight sulfur compounds and ASD is of particular interest, mainly due to due to the presence of the HTL that can lead to pathological changes [22]. The differences between the levels of HTL, Cys, and CysGly in the urine of both autistic and non-autistic children are presented. In this study, liquid chromatography with spectrofluorometric detection was used to determine HTL, and Cys and CysGly were determined with the use of the liquid chromatography with spectrophotometric detection (DAD). To the best of our knowledge, HTL has not yet been determined in the urine of autistic children.

## 2. Results

A validated HPLC method was applied to determine the levels of HTL, Cys, and CysGly in the urine of autistic and non-autistic children. The results were calculated as a ratio of the analyte of interest and urinary creatinine concentration in the unit μmol/mmol of creatinine. Individual differences in the concentrations of HTL, Cys, and CysGly between the two groups (autistic and non-autistic) were found (Table 1). The data published on The Human Metabolome Database [23] were checked, and information about the reference values in urine only for two compounds, Cys and CysGly, was found. Now, this is the first time the results for HTL in the urine of autistic children have been presented. The assessed levels of the determined compounds were compared with available reference values. 

**Figure 1:**
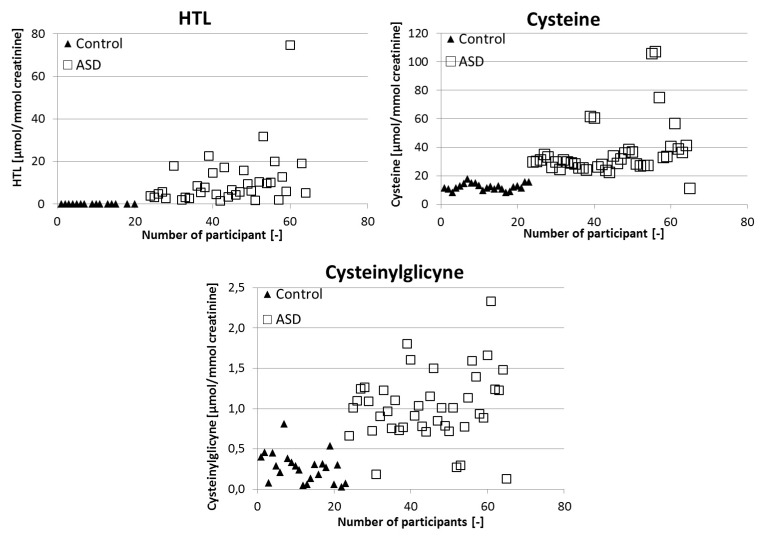
Individual differences in the levels of homocysteine thiolactone, cysteine, and cysteinylglycine between autistic and non-autistic children.

Significant differences in the mean and median are observed in the studied groups. The median and mean value for Cys in the ASD group fall within the reference range, but in the control group the value is below the reference range. In the case of CysGly, the median and mean value in the ASD group are above the reference range and in the control group it is below the reference range. The obtained results were characterized by high variability. The distribution of the resulting urinary HTL, Cys, and CysGly for the autistic children and potentially healthy children is displayed in Figure 1.

The levels of HTL, Cys, and CysGly were compared between the two groups using a Mann–Whitney U test, as shown in Table 2. A difference in the p-value of <0.05 is statistically significant. Statistical data shows that the children with autism were characterized by a statistically significantly higher level of HTL [p = 5.86 × 10^−8^], Cys [p = 1.49 × 10^−10^], and CysGly [p = 1.06 × 10^−8^] in urine compared with the control group.

**Figure 2:**
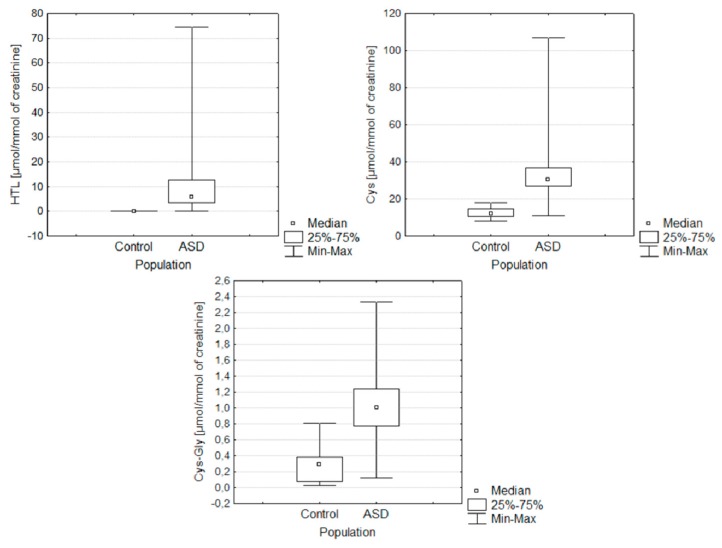
Box and Whisker plots for the compounds determined in the following groups: ASD and control. In these box plots, medians (not means) inside the 25–75% IQR are presented.

**Figure 3:**
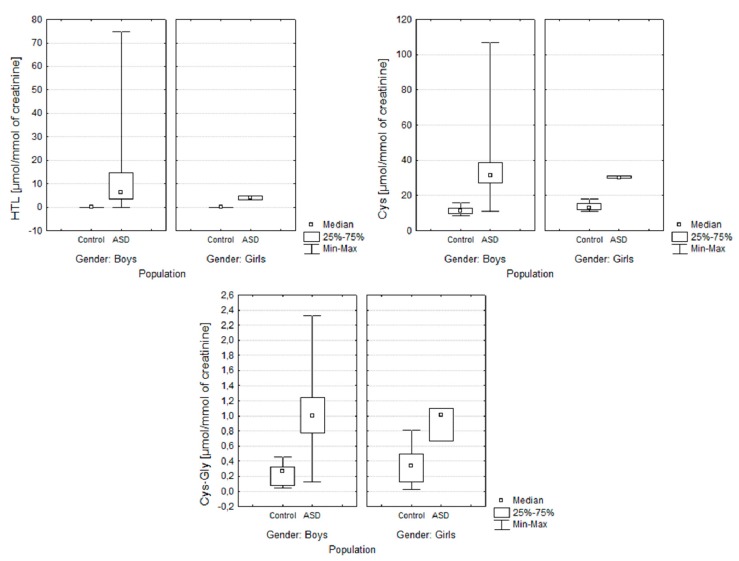
Box and Whisker plots for the compounds determined in the following groups: ASD and control gender-categorised. In these box plots, medians (not means) inside the 25–75% IQR are presented.

Figure 2 and Figure 3 present the distribution of the results obtained in both of the examined groups: ASD and control. They allow for the simultaneous evaluation of the differences in the content of sulfur compounds determined by gender and the study group.

Figure 3 presents the distribution of the obtained results in the groups of autistic and control children categorised in terms of gender. Application of the Shapiro–Wilk test showed that the hypothesis that the data was normally distributed could be rejected [p < 0.05]. Individual differences in the levels of sulfur compounds between the two groups (autistic children and non-autistic children) were found after performing the Mann–Whitney U test. The application of the Mann–Whitney U test test showed a significant difference between the examined groups. The difference in the p-value of <0.05 is statistically significant. The application of the Mann-Whitney U test showed a difference in the level of HTL [p < 0.05] and no difference between the levels of Cys and CysGly in autistic boys and girls. The gender of patients in this case does not influence the concentrations of sulfur compounds Cys [p = 0.08] and CysGly [p = 0.07].

Finally, the statistical analysis showed a positive correlation between the concentration of Cys and CysGly (correlation value ASD = 0.55, control = 0.51), and a slightly weaker correlation between HTL and CysGly (a positive correlation value in the ASD group 0.27, a negative correlation value in the control group 0.41).

**Figure 4:**
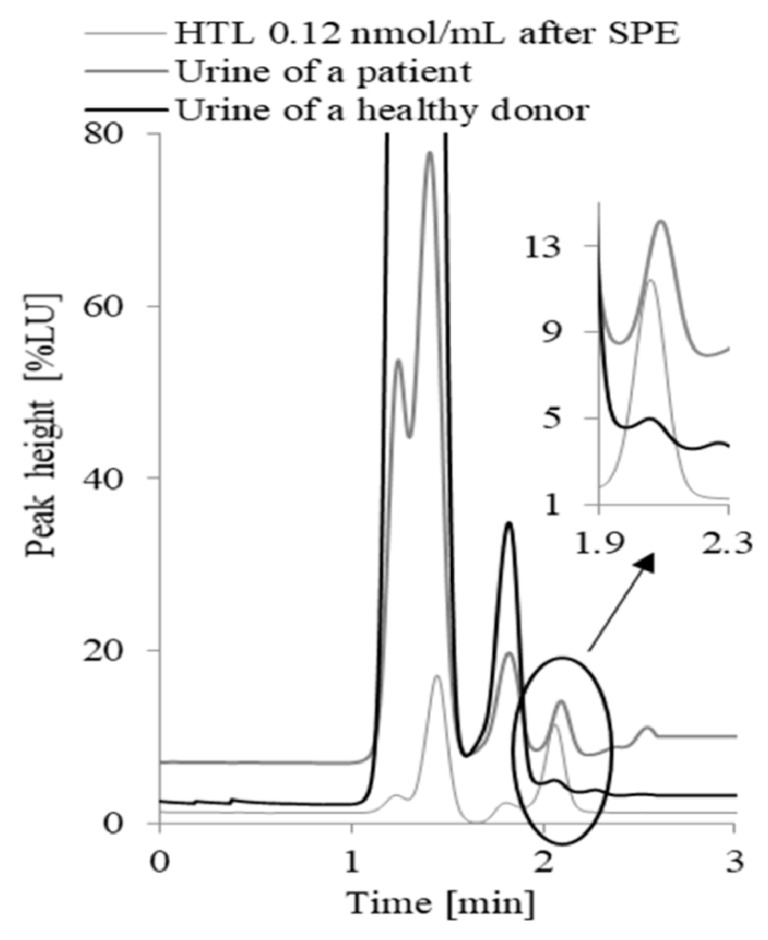
Typical chromatograms of a standard solution of HTL (0.12 nmol·mL^−1^) after the complete protocol of the solid phase extraction dedicated to urine samples, and HTL in the urine samples collected from a non-autistic child and a child with autism.

**Figure 5:**
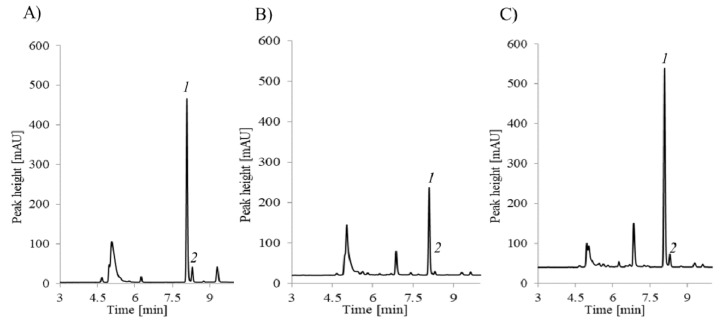
Typical chromatograms of a standard solution of Cys-1 and CysGly-2 (**A**) and the urine samples collected from a non-autistic child (**B**) and a child with autism (**C**).

Typical chromatograms of the analytes in the samples collected from a non-autistic child and a child with autism are presented in Figure 4 and Figure 5.

## 3. Discussion

A very interesting object of biological fluid analysis is urine due to the non-invasive sampling method, particularly in children. Thiols have been of great interest to researchers for years due to their important role in biological processes. The analysis of thiols plays a key role in the diagnosis of several diseases. Changes in the amounts of thiols in body fluids are observed when the cellular processes are disturbed. Amino acids containing sulfur contribute to the maintenance and integrity of cellular systems. This is possiblly due to the cellular capacity to detoxify toxic compounds that influence the cellular redox state, reactive oxygen species, and free radicals. Cys and CysGly are the main thiols identified in urine [24,25].

The concentration levels of HTL, Cys, and CysGly were statistically significantly higher in ASD individuals than in the control group. Individual differences in the level of HTL were observed in autistic boys and girls but no differences between the levels of Cys and CysGly were found. The gender of the study patients in this case does not influence the concentrations of the sulfur compounds of our interest.

In the literature, there is a lot of information about risk factors of neurodegenerative and cardiovascular diseases and neural tube defects caused by an elevated level of Hcy. Dietary protein methionine is the only known source of Hcy in the human body. Moreover, studies show that methionine ingested in excess is the most toxic amino acid [26,27]. Fau et al. reported that animals fed high-methionine diets for 2 years developed hyperhomocysteinemia and evidence of vascular disease [28]. Several recent studies have focused on the transsulfuration pathway in ASD, which suggested that children with ASD had abnormal levels of Hcy, Cys, and glutathione [29,30,31]. Hcy can be metabolized via two pathways: transsulfuration to Cys or remethylation to methionine. A defect in either of these pathways can cause an accumulation of Hcy. The process of tranassulfuration involves vitamin B6, whereas remethylation involves folic acid and vitamin B12. To lower the levels of Hcy, vitamins B6, B12, and folic acid are necessary. Improper dietary intakes of these nutrients can lead to elevated levels of Hcy [32]. According to Williams et al. (2005), nutritional deficiencies in folic acid and vitamins B6 and B12 result in higher levels of Hcy and an increase in some autistic symptoms [33]. The relationship between the effectiveness of dietary supplements of vitamin B6 in autism spectrum disorders was also presented by Adams et al. (2004). Their studies indicate a statistically significant improvement in sleep and gastrointestinal problems in ASD children compared with the placebo ones [34]. Xia (2011) presented the research on a 9-year-old boy with autism who responded positively to vitamin B6 nutritional supplements, which resulted in the improvement of sociability, communication, behavior, and cognitive awareness. The presented studies suggest that there is an important relationship between deficiencies in folic acid and vitamin B, and high levels of Hcy for the proper functioning of ASD children [35,36].

The transsulfuration pathway begins with Hcy. Cystathionine synthase irreversibly removed Hcy from the methionine cycle. This reaction initiates the transsulfuration pathway for the synthesis of sulfate, taurine, glutathione, and Cys [37]. Hcy is metabolized to HTL by methionyl-tRNA synthetase. This transformation contributes to Hcy toxicity and takes place as a result of an error-editing reaction during protein biosynthesis when Hcy is mistakenly replaced with methionine. Moreover, HTL reacts with the proteins modifying them, which causes their damage. Such modification is carried out by forming adducts—Hcy is *N*-linked to the ε-amino group of protein lysine residues. This causes impairment or a change in the protein function and induces an autoimmune response. In serum and human cells, an increased level of Hcy leads to elevated levels of HTL. HTL produced in human body is cleared by urinary excretion [24,36,38,39,40,41,42]. The significantly increased concentration of HTL in the urine of autistic children may be caused by genetic defects of enzymes involved in the metabolism of methionine. When methionine synthase activity is inhibited by folate or vitamin B12 deprivation, almost all Hcy is converted into HTL. The metabolic conversion of Hcy into HTL, the reactivity of the HTL toward proteins, and the resulting protein damage may explain some pathologic consequences of elevated Hcy levels, including ASD. Genetic alterations of enzymes, such as cystathionine β-synthase (CBS) or methylenetetrahydrofolate reductase (MTHFR), affect the increase in HTL [36]. MTHFR is responsible for the conversion of 5,10-methylenetetrahydrofolate (MTHF) to 5-methyltetrahydrofolate (5MTHF) and regulates folate availability. Codon 677 of the MTHFR gene causes a decrease in the activity of the MTHFR enzyme by 60% and, more importantly, an increase in Hcy levels, particularly in patients with low levels of B vitamins. In patients with inactive versions of genes encoding MTHFR or CBS, elevated levels of serum HTL have been observed [43]. Other important genetic aspects are cystathionine β-synthase (CBS) gene mutations. The CBS enzyme catalyzes a reaction that permanently removes Hcy from the methionine pathway. Removal is carried out by diverting Hcy to the transsulfuration pathway, where vitamin B6 as a co-factor is needed. The literature suggested that there are associations between ASD and functional gene variants within the B vitamin-dependent folate, methionine, and transmethylation pathways [44,45]. Low levels of 5MTHF in cerebrospinal fluids were observed in the study conducted by Ramaekers et al. (2002) [46]. Supplementation with folic acid eliminated the symptoms of neurological regression in children, while Schmidt et al. (2011) indicated that children whose mothers have MTHFR 677 TT, CBS genotypes and do not take prenatal vitamins have an increased risk of autism disorder [47].

Other main thiols identified in urine are Cys and CysGly. Cys, through the presence of a thiol group, has a high affinity for heavy metals and proteins containing Cys, which bind metals such as lead, mercury, and cadmium. Cys has antioxidant properties due to its ability to undergo redox reactions. Disorders of Cys metabolism lead to the high concentration of Cys in urine because the reabsorption mechanism in the kidneys does not function properly. Cys may be essential for individuals with a metabolic disease or patients with malabsorption syndormes. Because of the low concentration of Cys compared with glycine and glutamate, Cys limits amino acid for glutathione synthesis [48]. Bitar et al. (2018) reported changes in the concentration of metabolites related to oxidative stress, including glutathione metabolism, and also changes in methionine, Cys, arginine, and proline metabolism pathways in the urine of 40 ASD individuals [3]. A low level of Cys was found in young ASD children and a high level in older children (older than five years). Cysteine abnormalities are consistent with the impaired sulfation and metallothione in synthesis in ASD individuals [49].

CysGly, which is derived from the breakdown of glutathione, is a naturally occurring dipeptid. In plasma, CysGly is in a reduced, oxidized and protein-bound form. It interacts via redox and disulphide exchange reactions in a dynamic system. One of the functions of CysGly is a prooxidant that reduces ferric iron to ferrous iron [50]. Kurochkin et al. (2019) observed that within the glutathione pathway, in addition to the decrease in the glutathione intensity, two metabolites, such as L-cysteinylglycine and L-γ-glutamyl-L-cysteine, display intensity differences in ASD [51]. Enzymes catalyzing reactions involving glutathione, L-cysteinylglycine, and L-γ-glutamyl-L-cysteine were shown to contain genetic variants connected with ASD (polymorphisms in genes such as GPX1, GSTM1, GGT1, and GSS51–53). Additionally, in children with ASD, altered urinary levels of some free amino acids, including lowered levels of glutamate and higher glycine, alanine, and taurine, were reported. The researcher suggested that the changes might result from perturbation in sulfur-dependent detoxification and amino acid metabolism in ASD individuals [52]. In children with ASD, a low sulphation capacity is observed. Scientists suggested that ASD children were unable to effectively metabolise phenolic amines functioning as neurotransmitters, such as dopamine, serotonin, and tyramine [53]. The research conducted by James et al. (2009) indicated that pretreatment with B12 and folinic acid caused a change in metabolite concentration in ASD individuals compared with the control group. The researcher observed significant increases in Cys, CysGly, and glutathione concentrations. The improvements in transmethylation metabolites and the glutathione redox status observed after treatment suggest that targeted nutritional intervention with B12 and folinic acid might be of clinical benefit to some children with ASD [54].

In humans, an increased flow through the HTL pathway is observed when the transsulfuration or remethylation reaction is impaired by the genetic alteration of enzymes involved in Hcy metabolism (CBS or MTHFR), and/or folate metabolism, and/or by nutritional disorders, such as a limited B-vitamin supply or a high-methionine diet. The literature shows that HTL is associated with pathological conditions. Urinary HTL level is elevated under conditions predisposing neurological abnormalities, such as those caused by genetic CBS or MTHFR deficiencies. Among others, these may explain the role of HTL in ASD. The results of the study showed some limitations due to a small population of the study group and the fact that the dietary/medicinal intake of sulfur compounds cannot be excluded in study groups. Urinary low molecular weight sulfur compounds and HTL were determined in autistic children and, compared with the controls, indicate metabolic disturbances in autism. In conclusion, the crucial role that the elevated levels of HTL, Cys, and CysGly play in the pathogenesis of different diseases makes these compounds interesting targets for future investigations. This knowledge could help to explain the etiology of disorders and to assist in early diagnosis, as well as in the choice of different forms of intervention. It could also provide support in the treatment and prognosis of the disorders.

## 4. Materials and Methods

### 4.1. Patients and Sample Collection

This study included a total of 41 children with ASD, aged 3 to 17, and 24 non-autistic volunteers, aged 3 to 17. The specific characteristics of the studied population are presented in Table 3. The autistic children were not on a gluten-free, casein-free, or sugar-free diet. Result bias due to the dietary/medicinal intake of sulfur compounds cannot be excluded.

The first morning urine samples from autistic children who were assessed and diagnosed by clinicians specializing in the diagnosis and management of ASD children from the Center for Diagnosis and Therapy of Autism in Łódź Navicula (Poland), and non-autistic children were collected. The urine was collected and stored at −20 °C until analysis. This study was restricted to children with an ASD diagnosis in compliance with the criteria detailed in *The International Classification of Diseases* [55]. The study was approved by the local research ethics committee (Bioethical Commission at the Institute Polish Mother’s Health Center, Lodz, No. 30/2018), and the work was conducted in accordance with the Declaration of Helsinki.

### 4.2. Preparation of Urine Samples

#### 4.2.1. Sample Preparation for Urinary HTL

A urine sample (150 µL) was mixed with a sodium phosphate buffer [100 µL, 0.1 mol·L^−1^ (pH 7.8)], and 200 µL of the mixture was transferred to Strata C18-E (55 mm, 50 mg/mL) solid-phase extraction (SPE) cartridges (Phenomenex, Torrance, CA, USA). The SPE cartridges were solvated with 1.0 mL of 2-propanol, followed by 500 µL 0.1 molL^−1^ sodium phosphate buffer (pH 7.8). After loading 200 µL of the sample, the cartridges were washed with 250 µL 0.02 mol·L^−1^ HCl in a 50% methanol solution to remove any unwanted material. HTL was eluted with 150 µL 0.02 M HCl in a 70% acetonitrile–water solution, and 20 µL of the eluate was loaded onto a C18 reversed-phase HPLC column (Hamilton PRP-1 Column, 150 × 4.6 mm, 5 µm; Energy Way, Reno, NV, USA). The column was eluted isocratically with a solution containing 0.1 mol·L^−1^ NaOH, 0.01 mol·L^−1^ *O*-phthaldialdehyde, and 30% acetonitrile at a flow rate of 1 mlmin^−1^.

#### 4.2.2. Sample Preparation for Urinary Cys and CysGly

For the determination of Cys and CysGly in urine samples, the assay previously used to determine plasma aminothiols [18] was adopted. A total of 200 µL of urine was transferred to the polypropylene vial and diluted with 100 µL of 0.2 mol·L^−1^ pH 7.8 phosphate buffer. Disulfide bonds were reduced by treatment with 5 μL of 0.25 mol·L^−1^ tris(2-carboxyethyl)phosphine solution for 10 min at room temperature. Then, 5 µL of 0.1 mol·L^−1^ 2-chloro-1-methylquinolinium tetrafluoroborate solution was added and the action mixture was vigorously mixed before being kept for 3 min at 25 °C. Next, 50 µL of 3 mol·L^−1^ perchloric acid was added. A total of 10 µL of the mixture was loaded onto a C18 reversed-phase HPLC column Aeris PEPTIDEXB-C18 (150 mm, 4.6 mm, 3.6 mm) column from Phenomenex.

### 4.3. Analytical Methods

The compounds were determined by simple, rapid, and validated high-performance liquid chromatography methods described in detail in [19] for HTL and in [17] for Cys and CysGly, respectively. The LOQ values for CysGly and Cys were 0.12 and 0.08 μmol L^−1^, respectively. In the case of HTL, the LOQ was 0.02 μmol L^−1^. Linearity in the detector response for total thiols was observed over the range of 5–300 μmol L^−1^ for Cys and 0.25–50 μmol L^−1^ for CysGly.

A 1100 Series HPLC instrument (Hewlett–Packard Waldbronn, Germany) containing a quaternary pump, autosampler, thermostated column compartment, vacuum degasser, and the spectrofluorimetric detector 1200 series was used to determine HTL. For the instrument control, data acquisition, and analysis, a Hewlett–Packard ChemStation for LC 3D system, including a single instrument ChemStation software, was used. For extraction, SPE cartridges (Strata C 18 U, Phenomenex, CA, USA) were used.

The separation of HTL was achieved in 3 min. The urine sample was injected onto a reversed-phase Hamilton PRP-1 column (150 × 4.6 mm, 5 µm; Energy Way, Reno, NV, USA). The column was eluted isocratically at 25 °C with a mobile phase containing 0.1 mol·L^−1^ NaOH, 0.01 mol·L^−1^ OPA, and 30% acetonitrile at a flow rate of 1 mL·min^−1^. Under these conditions, HTL detected and quantified by fluorescence using excitation at 370 nm and emission at 480 nm elutes as a sharp peak at 2.1 min. In the case of CysGly and Cys, 2-chloro-1-methylquinolinium tetrafluoroborate (CMQT) was used as a derivatization reagent. The separation of the analytes was achieved in 12 min using HPLC with UV detection at 355 nm. The analyses were performed on a 1220 Infinity LC system (Agilent Technologies, Waldbronn, Germany) equipped with a binary pump that was integrated with a two-channel degasser, autosampler, column oven, and a diode-array detector (DAD) controlled by Open LAB CDS Chem Station software. Chromatographic separation was accomplished on an Aeris PEPTIDEXB-C18 (150 mm, 4.6 mm, 3.6 mm) column from Phenomenex with a gradient elution (0–6 min 7–62% B, 6–8 min 62–70% B, 8–12 min 70% B), a mobile phase ((A) acetonitrile and (B) 0.2% trichloroacetic acid, pH 2.25), and a flow rate 1.0 mL·min^−1^. For the column recondition, 1 min post time was used.

### 4.4. Urinary Creatinine Determination

In clinical medicine, one of the most widely used and commonly accepted tests of renal function is the renal clearance of creatinine [56]. The renal clearance of creatinine is based on the axiom of the rate of a substance removing from the plasma by the kidney and must be equal to the rate of its excretion into the urine [57]. Blood and urine tests of creatinine show the quality of the kidneys’ function and also can help to control the pharmacokinetics of drugs. Thus, literature data clearly indicate that creatinine is recognized as the reference compound with the lowest variance, both within and between individuals [58,59]. For the estimation of creatinine in the urine samples, the previously elaborated and thoroughly validated method was exploited [60].

### 4.5. Statistical Analysis

The Statistica 9.0 software (StatSoft, Polska STATISTICA, version 9.0, Quest Software, Aliso Viejo, CA, USA) was used for statistical analysis. The normal distribution of the data was tested using the Shapiro–Wilk method. The non-normal data was analyzed with a nonparametric Mann–Whitney U test to determine the differences between the values of particular sulfur compounds in groups. The results were considered statistically significant when p < 0.05.

## Figures and Tables

**Table 1:** Values obtained for homocysteine thiolactone, cysteine, and cysteinylglycine (μmol/mmol of creatinine) in the urine samples of the entire tested population.

Name of Compound	Population	Mean	Conf. −95%	Conf. +95%	Median	Min.	Max.	Lower Quartile	Upper Quartile	Variance	Std.Dev.	Standard Error
**Homocysteine thiolactone**	ASD	10.3024	5.9807	14.6241	5.8872	0.0024	74.6099	3.3839	12.7344	168.0128	12.9620	2.1309
	Control	0.0079	0.0028	0.0130	0.0041	0.0003	0.0342	0.0011	0.0126	0.0001	0.0092	0.0024
**Cysteine**	ASD	36.8925	30.8243	42.9608	30.6270	11.1375	107.502	27.0894	36.8070	379.1978	19.4730	3.0047
	Control	12.3378	11.2617	13.4139	12.1620	8.3642	17.9055	10.7592	14.7696	6.1924	2.4885	0.5189
**Cysteinylglycine**	ASD	1.0229	0.8877	1.1582	1.0070	0.1291	2.3290	0.7689	1.2367	0.1884	0.4340	0.0670
	Control	0.2734	0.1924	0.3543	0.2920	0.0293	0.8082	0.0795	0.3847	0.0350	0.1872	0.0390

**Table 2:** Concentration of homocysteine thiolactone, cysteine, and cysteinylglycine in the urine of autistic children (ASD) and non-autistic children (non-ASD) (M ± SD) (μmol/mmol of creatinine).

Name of Compound	ASD	non-ASD	ASD vs non-ASD
Homocysteine thiolactone	10.30 ± 2.13	7.90 ± 2.4×10^−3^	Z = −5.42, p = 5.86×10^−8^
Cysteine	36.89 ± 3.00	12.34 ± 0.52	Z = −6.41, p = 1.49×10^−10^
Cysteinylglycine	1.02 ± 0.07	0.27 ± 0.04	Z = −5.72, p = 1.06×10^−8^

**Table 3:** Characteristics of participants.

	ASD Group	Control Group
Total participants	41	24
Male	38 (92.7%)	16 (66.7%)
Female	3 (7.3%)	8 (33.3%)

## Abbreviations

ASD	autism spectrum disorder
BMI	body mass index
CBS	cystathionine β-synthase
CE	capillary electrophoresis
CMQT	2-chloro-1-methylquinolinium tetrafluoroborate
Cys	cysteine
CysGly	cysteinylglycine
DAD	diode-array detector
γ-GluCys	γ-glutamylcysteine
GSH	glutathione
Hcy	homocysteine
HTL	homocysteine thiolactone
IQR	interquartile range
LOD	limit of detection
LOQ	limit of quantitation
MTHFR	methylenetetrahydrofolate reductase
RP-HPLC/UV–Vis	high-performance reverse phase liquid chromatography with spectrophotometric detection
SD	standard deviation
TCEP	tris(2-carboxyethyl)phosphine
UV	ultra-violet detection
